# Home Spirometry in Children with Cystic Fibrosis

**DOI:** 10.3390/bioengineering10020242

**Published:** 2023-02-11

**Authors:** Ariel Berlinski, Pamela Leisenring, Lauren Willis, Sandra King

**Affiliations:** 1 Division of Pulmonology and Sleep Medicine, Department of Pediatrics, University of Arkansas for Medical Sciences, Little Rock, AR 72202, USA; 2 Arkansas Children’s Hospital Cystic Fibrosis Care Center, Little Rock, AR 72202, USA

**Keywords:** cystic fibrosis, home spirometry, pediatrics, FEV_1_, telehealth

## Abstract

We report the implementation of a pediatric home spirometry program at our institution. A respiratory therapist provided either a virtual or an in-person initiation visit that included a coached spirometry session. Families were instructed to perform daily uncoached spirometry sessions for 5 days. The program’s quality assurance component was deemed not to be human research by the local IRB. In total, 52 subjects completed an initiation visit (34 with at least 3 additional uncoached spirometry sessions). The clinic spirometry and coached (same-day) sessions and uncoached (same-week) sessions were completed by 12 and 17 subjects, respectively. The median (99% CI) coefficients of variation for FEV_1_% of the uncoached maneuvers were 3.5% (2.9–5.9%). The median (IQR) FEV_1_% and FEV_1_ (mL) absolute differences between coached and uncoached home spirometry were −2% (−4 and +3%) and −25 mL (−93 and +93 mL), respectively. The median (IQR) absolute differences in FEV_1_% and FEV_1_ (mL) between coached or uncoached home spirometry and clinic spirometry were −6% (−10 and −2%) and −155 mL (−275 and −88 mL), and −4% (−10 and +5%), and −110 mL (−280 and +9 mL), respectively. Differences in absolute FEV_1_ (L) and FEV_1_% were found among different modalities of spirometry performed by people with cystic fibrosis. Understanding the variability of uncoached home spirometry and the differences among coached and uncoached home spirometry, hospital and coached home spirometry, and hospital and uncoached home spirometry for any given individual is crucial to effectively utilize this tool in clinical care.

## 1. Introduction

Cystic fibrosis (CF) is a genetic condition resulting in the abnormal function of the cystic fibrosis transmembrane regulator protein [1]. This defect results in chronic health problems involving the lungs, intestines, and other organs and systems. Mortality is generally related to the deterioration of lung function that results from a chronic cycle of infection and inflammation. Therefore, monitoring of the lung function is at the core of maintaining lung health in people with cystic fibrosis [2]. Spirometry is performed at each clinical visit and pediatric patients 6 years or older are typically able to perform spirometry. Changes in the forced expiratory volume in the first second (FEV_1_) are used to help define a pulmonary exacerbation, determine the length of antibiotic therapy, suspect cystic fibrosis-related diabetes, evaluate the response to different medications including modulator therapy, and decide the timing for referral to a lung transplantation program [3,4,5]. In general, a 10% variation in FEV_1_ percent predictive is the threshold used in clinical practice.

Although spirometry is routinely performed in clinics, home spirometry has been used to monitor lung function for the early diagnosis of bronchiolitis obliterans syndrome in people with cystic fibrosis who had undergone lung transplantation [6]. Some care centers previously explored the potential use of home spirometry for routine monitoring of lung health in people with cystic fibrosis [7,8,9,10]. However, device availability, cost, software limitations, and technological limitations hindered its widespread adoption. The COVID-19 pandemic imposed a shift from in-person to telehealth care [11]. This change in practice resulted in greater interest in using home spirometry. At the same time the device cost had decreased, creating an opportunity for change. The Cystic Fibrosis Foundation responded to the need for continued monitoring of lung function despite low in-person clinic attendance by providing home spirometers to people with cystic fibrosis and accredited care centers [12]. In addition to monitoring of lung function in cystic fibrosis, home spirometry has been used to monitor lung function in a variety of conditions, including asthma, interstitial lung diseases, idiopathic pulmonary fibrosis, and Duchenne muscular dystrophy, as well as the evaluation of the sequela of respiratory infections such as COVID-19, sarcoidosis, post-bone marrow transplantation, and others [13,14,15,16,17,18,19,20,21,22,23,24]. The use of this technology has the potential for more frequent testing without the need for patients and families to travel to medical centers. This not only has health implications but financial implications as well, such as reducing transportation expenses and time off work. However, there are no clear guidelines on how to implement the use of home spirometry in children. It is also unclear how to optimally utilize this technology (daily, weekly, monthly, with symptoms, etc.).

Most previously published studies in children with cystic fibrosis were conducted using uncoached spirometry. Clinical experience from performing spirometry in the clinic/hospital setting underscores the importance of proper coaching for obtaining maximal efforts in children. Thus, it is relevant to investigate if there are differences in FEV_1_ when pediatric patients are coached or perform the maneuvers by themselves (uncoached spirometry). Previous pediatric studies reported bias between home spirometry and clinic spirometry. During clinic spirometry, the patient will tidal breathe before taking a deep breath to total lung capacity and then forcefully exhale until reaching residual volume. However, some home spirometers do not allow tidal breathing before full inspiration. These devices require the subject to adequately coordinate connecting to the mouthpiece and do not allow for errors in the coordination often displayed in children. Therefore, specific training is required to master the performance of home spirometry. Understanding several aspects of their performance before incorporating home spirometry into clinical care is crucial [8,25,26,27,28,29,30]. Firstly, it is necessary to know the degree of agreement between FEV_1_ obtained during clinic spirometry and home spirometry session with and without coaching. Secondly, it is important to know the degree of agreement between coached and uncoached FEV_1_ values. Thirdly, it is important to know the degree of variability of the FEV_1_ obtained during an uncoached home spirometry session.

Once we received the home spirometry devices, our institution developed a program to implement their use in people with cystic fibrosis. Aware of previously published data, we incorporated a quality assurance component designed to help us understand the performance of the home spirometer.

## 2. Methods

**Figure 1:**
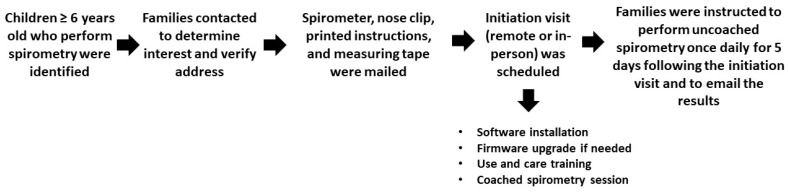
Timeline of protocol.

**Figure 2:**
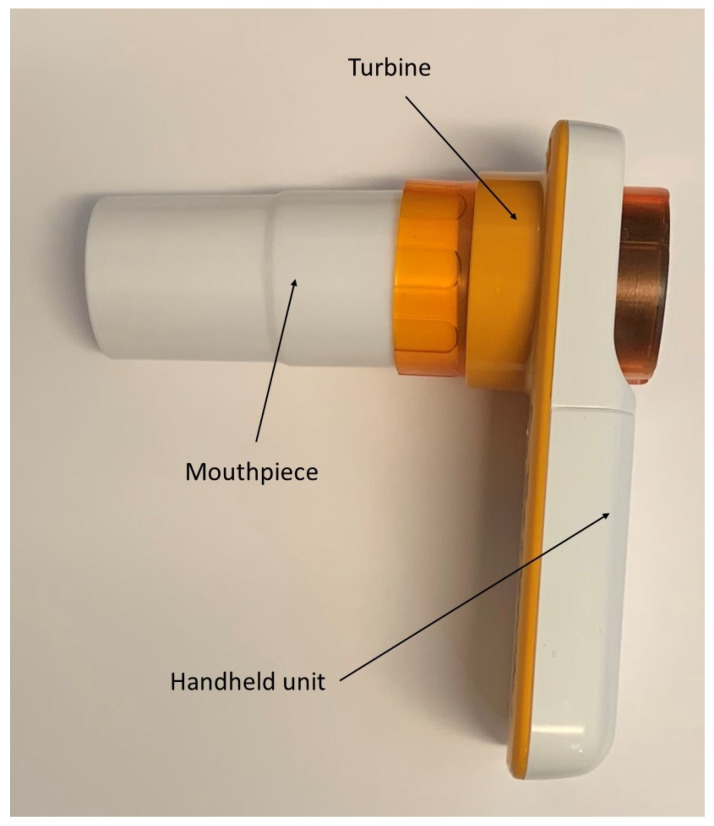
ZephyRx MIR Spirobank Smart spirometer with reusable turbine and mouthpiece.

This program was implemented at the Pediatric Cystic Fibrosis Care Center at Arkansas Children’s Hospital, the only pediatric care center in the state of Arkansas accredited by the Cystic Fibrosis Foundation. The local institutional review board (University of Arkansas for Medical Sciences, Little Rock, AR, USA) deemed this activity not to constitute human research. The program was developed in collaboration between the Care Center, the Respiratory Care Department, and the Pulmonary Diagnostic Laboratory (Figure 1). Firstly, the families of people with cystic fibrosis 6 years or older who had previously been able to successfully perform spirometry were identified and contacted to verify their interest in participating in the home spirometry program and a delivery address. The families who agreed to have access to home spirometry were mailed a spirometry unit, nose clips, a printed copy of updated hardware and software instructions, and a measuring tape for height measurement at home, in case a significant delay between the clinic and home spirometry occurred. The latter was performed in case too much time elapsed between the 2 different measurements [31]. An appointment was scheduled with a designated respiratory therapist to conduct either a virtual (required 2 devices: one for spirometry and the other to provide real-time coaching) or an in-person standardized initiation visit. The initiation visit typically lasted 45–60 min. However, the virtual initiation often required some additional time due to connectivity issues. Only 2 respiratory therapists, who followed a checklist (Checklist S1 in Supplementary Materials), were assigned to the program to provide consistency. In-person initiation visits were followed up by remote communications to troubleshoot any connectivity problems. During the initiation visit, the device was set up and a software installation and firmware upgrade were conducted if needed. The child and family were trained on how to use and care for the home spirometer. The differences in technique between the maneuver they were used to (tidal breathing before maximal inhalation) and the maneuver required for the home spirometry were explained and demonstrated by patients. This was followed by children performing a coached home spirometry session. The latter was completed when either acceptability/reproducibility were achieved or 8 spirometric maneuvers were completed. During coached maneuvers, the respiratory therapist observed the child performing the maneuver and provided verbal instructions regarding the timing of different steps. Specifically, the respiratory therapist encouraged the child to inhale deeply and forcefully exhale as long as possible, consistent with a spirometry performed at the clinic. The families were instructed to have the child perform daily uncoached spirometry for 5 days following the initiation visit. No reminders were sent by either the respiratory therapist or the spirometry software. The ZephyRx MIR Spirobank Smart Spirometer (Troy, NY, USA) with a reusable turbine and mouthpiece (Figure 2) provided by the Cystic Fibrosis Foundation was utilized for the home spirometry program. This device used the usability and acceptability criteria established by the 2019 American Thoracic Guidelines [32]. The devices were connected to a web-based dashboard where spirometry data were stored. The use of the dashboard was initially free, but the company later changed to a usage-fee model. At the time these data were obtained, the home spirometer did not allow in-app coaching, but this feature was added in later software versions. Families were instructed to email results to the Pulmonary Diagnostic Laboratory through the spirometry software. This was conducted in case we were not able to use the dashboard in the future. While uploading the data to the dashboard occurred automatically, emailing the spirometry data required an extra step from the participants. Global Lung Initiative reference equations were used to determine the percent predictive values [33].

The following demographic data were collected: age, sex, ethnicity, weight (kg), and height (cm). The following spirometric data obtained from the best maneuver were collected: forced vital capacity (FVC) absolute value and percent predictive, FEV_1_ (absolute value and percent predictive), FEV_1_/FVC, and forced expiratory time (FET).

Adherence to the process was evaluated by the number of subjects who completed 4 or more uncoached home spirometry sessions and by the number of families emailing the results as instructed. The repeatability of FEV_1_ obtained with the uncoached maneuver was evaluated by calculating its coefficient of variability. Dichotomous variables were compared with chi-square statistics. Continuous variables were compared with an unpaired *t*-test with unequal variances. Bland-Altman plots were used to compare the FEV_1_ data obtained during coached and uncoached home spirometry, during coached spirometry and clinic spirometry performed the same day, and during uncoached home spirometry and clinic spirometry performed within 1 week of the first uncoached session [34,35]. A statistical software Prism 9.0 (GraphPad Software, San Diego, CA, USA) was used.

## 3. Results

### 3.1. Coached Spirometry

In total, 52 subjects completed a home spirometry initiation visit between 6–19–20 and 12–21–20. Demographic data for the participants are reported in Table 1.

The median (99% CI) FVC absolute value for the coached maneuvers was 2.77 L (2.17–3.62 L). The median (99% CI) FVC percent predictive for the coached maneuvers was 108% (102–118%). The median (99% CI) FEV_1_ absolute value for the coached maneuver was 2.23 L (1.79–2.95 L). The median (99% CI) FEV_1_ percent predictive for the coached maneuvers was 100% (93–110%). The median (99% CI) FEV_1_\FVC for the coached maneuvers was 0.81 (0.78–0.84). The median (99% CI) FET for the coached maneuvers was 8.4 s (7.6–9.4 s).

### 3.2. Uncoached Spirometry

The subjects completed a median (99% CI) number of 4 (3–5) uncoached sessions following the initiation visit. Thirty-four subjects completed at least three uncoached spirometry sessions and were included in the coefficient of variation analysis. No differences in age (*p* = 0.88), sex (*p* = 0.56), ethnicity (*p* = 0.99), weight (*p* = 0.69), height (*p* = 0.46), and percent predictive FEV1 (*p* = 0.57) were found between those who completed 3 or less and those who completed more than 3 uncoached spirometry sessions (Table 1).

The following data refers to the 34 subjects who completed at least 3 uncoached spirometry sessions. The median (99% CI) FVC absolute value for the uncoached maneuver was 2.90 L (2.28–3.64 L). The median (99% CI) FVC percent predictive for the uncoached maneuver was 107% (102–119%). The median (99% CI) FEV_1_ absolute value for the uncoached maneuver was 2.48 L (1.80–2.96 L). The median (99% CI) FEV_1_ percent predictive for the uncoached maneuver was 102% (99.7–109.5%). The median (99% CI) FEV_1_\FVC for the uncoached maneuver was 0.81 (0.78–0.85). The median (99% CI) FET for the uncoached maneuver was 8.6 s (7.6–9.9 s).

The median (99% CI) coefficient of variation for FVC percent predictive for the uncoached maneuver was 2.8% (1.8–4.5%). Of note, 12% of the subjects had a coefficient of variation >10%. The median (99% CI) coefficient of variation for FEV_1_ percent predictive for the uncoached maneuver was 3.5% (2.9–5.9%). Of note, 18% of the subjects had a coefficient of variation >10%. The median (99% CI) coefficient of variation for FEV_1_/FVC for the uncoached maneuver was 2.7% (2.2–4.4%). Of note, 9% of the subjects had a coefficient of variation >10%. The median (99% CI) coefficient of variation for FET for the uncoached maneuver was 16.5 (9.4–21.6%). Of note, 32% of the subjects had a coefficient of variation <10%.

### 3.3. Paired Comparisons

The comparison of FEV_1_ values obtained during coached and uncoached home spirometry sessions, coached home spirometry and clinic spirometry sessions, and uncoached home spirometry and clinic spirometry sessions can be seen below.

**Figure 3:**
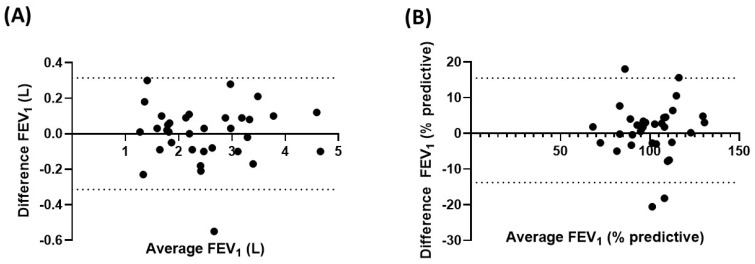
Bland-Altman plot for FEV_1_ obtained during coached and uncoached home spirometry sessions. FEV_1_ = forced expiratory volume in the first second; (**A**) absolute value (L); (**B**) percent predictive value.

Figure 3 shows a Bland-Altman plot comparing the absolute and percent predictive FEV_1_ values from 34 subjects obtained during the coached and uncoached home spirometry sessions. The median (IQR) FEV_1_ percent predictive value (%) and FEV_1_ absolute value (mL) absolute differences between the coached and uncoached home spirometry were −2% (−4 and +3%) and −25 mL (−93 and +93 mL), respectively. Of note, 15% of the subjects had an absolute difference in FEV_1_% ≥10%. There was no difference in bias across the spectrum of FEV_1_. Children who had less than three uncoached spirometry sessions had larger differences that those who performed more uncoached sessions. The median (IQR) FEV_1_ percent predictive value (%) and FEV_1_ absolute value (mL) absolute differences between coached and uncoached home spirometry were −2% (−2.5 and +8%) and −20 mL (−50 and +190 mL), respectively.

**Figure 4:**
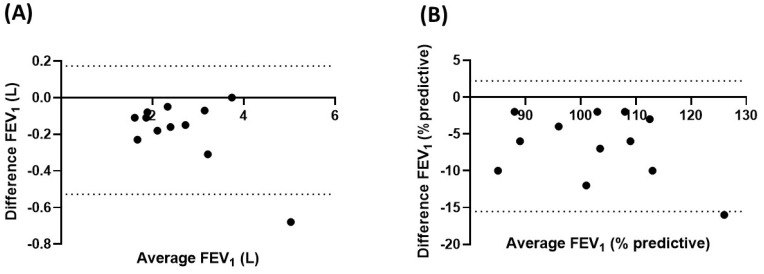
Bland-Altman plot for FEV_1_ obtained during coached home spirometry and clinic spirometry sessions. FEV1 = forced expiratory volume in the first second; (**A**) absolute value (L); (**B**) percent predictive value.

Figure 4 shows a Bland-Altman plot comparing the absolute and percent predictive FEV_1_ values obtained during the coached home spirometry and clinic spirometry sessions performed on the same day (12 subjects). The median (IQR) absolute differences in FEV_1_ predictive value (%) and FEV_1_ absolute value (mL) between coached home spirometry and hospital spirometry sessions were −6% (−10 and −2%) and −155 mL (−275 and −88 mL), respectively. However, 42% of the subjects had an absolute difference in FEV_1_% ≥10%. There was no difference in bias across the spectrum of FEV_1_.

**Figure 5:**
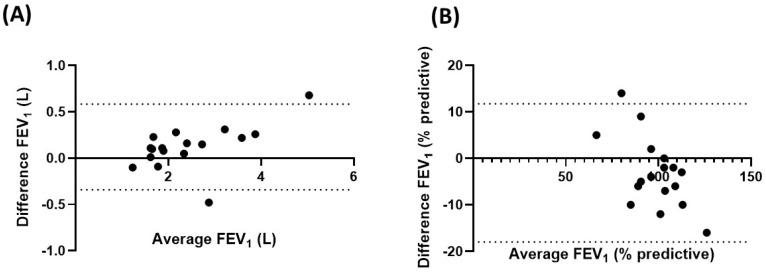
Bland-Altman plot for FEV_1_ obtained with uncoached home spirometry and clinic spirometry. FEV1 = forced expiratory volume in the first second; (**A**) absolute value (L); (**B**) percent predictive value.

Figure 5 shows a Bland-Altman plot comparing the absolute and percent predictive FEV_1_ values obtained during the uncoached home spirometry and clinic spirometry sessions performed within 1 week of the first uncoached session (17 subjects). The median (IQR) absolute differences in FEV_1_ predictive value (%) and FEV_1_ absolute value (mL) between coached home spirometry and hospital spirometry were −4% (−10 and +5%) and −110 mL (−280 and +9 mL), respectively. However, 24% of subjects had an absolute difference in FEV_1_% ≥10%. There was no difference in bias across the spectrum of FEV_1_.

### 3.4. Adherence

Approximately one-third of the subjects (18/52, 34.6%) did not complete the required 4 or more uncoached home spirometry sessions. This number includes 7 (13.5%) subjects who did not complete any uncoached home spirometry sessions. Only 44% of the participants who completed at least one uncoached home spirometry session (20/45) sent their spirometric data via email.

## 4. Discussion

We found clinically significant differences in FEV_1_ obtained during either coached or uncoached home spirometry compared with the clinic spirometry sessions. Home spirometry provided values that were lower than the ones obtained during clinic spirometry. Although the median bias was low, the confidence interval of the difference was wide. In addition, a significant number of subjects had differences 10% or greater, a threshold that is clinically used to determine deterioration/improvement in lung function. We also found that the bias in FEV_1_ values obtained with coached and uncoached home spirometry sessions was larger in those who performed less uncoached sessions. Adherence to the protocol was relatively low. Our data suggest that these differences have to be evaluated in order to incorporate the use of home spirometry into clinical care.

Comparisons with previous data obtained in children with cystic fibrosis are complex due to the differences in the home spirometry devices used [8,25,26,27,28,29,30]. Overall, however, the results showed a similar trend, with the exceptions noted below.

Bastian-Lee et al. compared clinic spirometry with home spirometry (VM Plus, Clement Clarke, Harlow, UK) in 70 children including 27 with CF [8]. They reported a coefficient of variation for FEV_1_ of 4.3% and limits of agreement for FEV_1_ of −3 to +190 mL. While the coefficient of variation was similar to ours, the limits of agreement were not. The home spirometry overestimated FEV_1_ when compared to the clinic spirometry. The difference in findings could be due to the different type of device used.

Avdimiretz et al. compared clinic and home spirometry (Micro Loop Spirometer, CareFusion, Yorba Linda, CA, USA) in 76 children with cystic fibrosis [25]. They reported a bias in FEV_1_ of −65 mL with large limits of agreements (+189 to −319 mL). Similar to us, they found a large number of children (15%) with home spirometry rendering FEV_1_ values ≥10% predictive than the clinic spirometry. They reported a similar coefficient of variation for the repeated measurement of FEV_1_ (6.7%).

Gerzon et al. studied 36 children with cystic fibrosis and compared clinic and home spirometry (AM2+, Carefusion, Houten, The Netherlands) [26]. Similar to us, they found a −180 mL bias with comparable limits of agreements of −8 to −270 mL.

Krizinga et al. evaluated a different hand-held turbine-based home spirometer (Air-Next, Stockholm, Sweden) in pediatric subjects with asthma and cystic fibrosis [27]. The bias in FEV_1_ (mL) between clinic and uncoached home spirometry averaged 40 mL. However, the limits of agreement were −270 mL to +352 mL. This is in contrast to our findings, which showed a larger bias (−110 mL) but narrower limits of agreement (−280 and +9 mL). We hypothesize that the differences could be attributed in part to the different software used by the two home spirometer brands.

Davis et al. compared the performance of two brands of home spirometers [NuvoAir (Boston, MA, USA) and ZephyRx MIR Spirobank Smart Spirometer (Troy, NY, USA)] for uncoached maneuvers in children with cystic fibrosis [28]. They reported a higher adherence than ours (80% versus 65.4 and 48%). This could be due in part to the fact that theirs was structured as a research protocol and ours as a quality assurance component of the implementation of the use of home spirometry. The motivation of the participants is likely to be higher in Davis et al.’s study. In addition, we used different definitions of adherence. Furthermore, while we looked at short-term adherence, they looked at long-term adherence to home spirometry. Similar to us, the feasibility study by Shakkottai reported a 60% adherence [9]. The lowest adherence of our report corresponds to the extra task required by the families to send the spirometry results via email. Davis et al. also reported a 5.1% and 8.5% lower FEV_1_ for NuvoAir and ZephyRx, when compared to a recent clinic spirometry. The bias in FEV_1_ in our study was slightly lower (−6 and −4% for coached and uncoached spirometries). This could be due in part to the fact that we compared clinic spirometry data that were closer to home spirometry than the study by Davis et al.

Doumit et al. compared FEV_1_ obtained with clinic spirometry and with in-person coached ultrasonic home spirometry (Spiromome, Inofab, Ankara, Turkey) in 59 children, including 49 with a diagnosis of cystic fibrosis [29]. They reported means (limits of agreement) of 10 mL (−220 to +240 mL) and −0.4% (−8.6 to 9.4%) for FEV_1_ absolute and percent predictive, respectively. Doumit et al. reported that 4 and 22% of the subjects had a bias greater than 10% and 150 mL, respectively. Our study had a larger number of subjects that had a 10% or greater bias in FEV_1_. We speculate that this difference could be in part due to hardware differences, mainly the use of turbine versus ultrasonic pneumotachometer.

This is the first report to compare in a single cohort home spirometry results using home spirometry with and without coaching. We were surprised that we did not see larger differences between the coached and uncoached maneuvers. We speculate that this was due to the fact that the uncoached home spirometries were performed during the days following the coached maneuvers. Therefore, the technique was still fresh in their minds. The durations of the maneuvers were quite similar, thus supporting this explanation. Previously, Fettes et al. reported that maneuvers obtained with coaching had better quality than those obtained during uncoached maneuvers [30]. Their study was conducted in two different cohorts. We agree with Fettes et al. that home spirometry should be performed in children during supervised sessions.

### Clinical Implications

The use of telehealth to deliver care in individuals with cystic fibrosis will continue. It is highly likely that it will be incorporated into the standard of care for certain groups of individuals who have more preserved lung and nutritional health. The patients with more advanced lung disease could also benefit from the use of this technology. A comparison of our and previously published data highlights that FEV_1_ results obtained with one brand/technology of home spirometry cannot be extrapolated to others. In addition, FEV_1_ results obtained with clinic spirometry and home spirometry are not interchangeable. Programs using home spirometry need to determine the bias between clinic and home spirometry. An important note is that those who performed a low number of uncoached spirometry sessions showed higher biases between the FEV_1_ obtained during coached and the uncoached spirometry sessions than those who performed more sessions. This technology might not be a choice for those with poor repeatability. The decision to use this technology should be conducted on an individual basis. However, this technology has the potential of avoiding hospital care, as shown in other conditions [36]. We also found that automated processes outperform those that require extra steps. Reminders could possibly enhance adherence to scheduled procedures [37,38]. This should be taken into account for future software developments. The proper instruction of the maneuvers is key to reducing the risk of adverse events related to the procedure and could be part of clinical risk monitoring activity [39].

This study has several limitations, including the small number of subjects and the lack of long-term follow-up. Future studies should investigate if the gap in FEV_1_ among different modalities changes over time. We also did not survey children and families with a low number of uncoached spirometries to try to understand their barriers. Although we recorded the number of sessions each subject performed, we did not record the number of maneuvers completed during each session. This report also has some strengths because it compared coached to uncoached home spirometry, adding new information to the growing body of literature. In addition, this was a real-life use of the device after specific instructions were provided to children and families. Newer studies evaluating the new in-app coaching feature for this device should be completed. Finally, new software needs to incorporate gamification to increase the interest and adherence of pediatrics users.

## 5. Conclusions

Differences in the absolute FEV_1_ (L) and FEV_1_ percent predictive were found among different modalities of spirometry performed by children with cystic fibrosis. Understanding the variability of uncoached home spirometry, as well as the differences among coached and uncoached home spirometry, hospital and coached home spirometry, and hospital and uncoached home spirometry for any given individual is crucial to effectively utilize this tool in clinical care.

## Figures and Tables

**Table 1:** Demographic information.

Subjects	n	Age (Years)	Sex (Male/Female)	Ethnicity (Caucasian)	Weight (kg)	Height (cm)
ALL	52	12.7 ± 4	30/22	48	45.2 ± 18.3	145 ± 18
<3 uncoached tests	18	12.6 ± 4.5	9/9	17	43.6 ± 23.3	142 ± 20
≥3 uncoached tests	34	13 ± 3.7	21/13	31	46 ± 15.3	146 ± 17
*p* value		0.88	0.56	0.99	0.69	0.46

## Data Availability

Data are contained in the article.

## Supplementary Materials

The following are available online at https://www.mdpi.com/article/10.3390/bioengineering10020242/s1, Checklist S1. Home spirometry initiation visit checklist.
