# Supplementary material for: Home Spirometry in Children with Cystic Fibrosis
Source: Bioengineering (Basel). 2023 Feb 11;10(2):242. doi: 10.3390/bioengineering10020242 (PMC9952128; doi:10.3390/bioengineering10020242)
Supplement: Supplementary file 1 [file bioengineering-10-00242-s001.zip › bioengineering-2133133-supplementary.pdf]

Home Spirometry Initiation Visit Checklist

Education Checklist for MIR Spirobank Smart spirometer:	
	Identify if patient is well and if there is an adult present if the patient is a minor. If sick, reschedule Home Initiation and instruct caregiver to call the Pulmonary office.
	Inform that device is single person use (don't have everyone try it)
	Review warranty and no replacement available if broken or lost (emphasize care)
	Instruct on putting device together (batteries, etc.)
	Download ZephyRX Breathe Easy APP or make sure the APP is updated.
	Review device set up and creating user profile. If existing, ensure current height in profile
	Do not block turbine. No moving overhead fan during test.
	Teach technique.
	Discuss how to email the data.
	Teach device cleaning and disinfecting. Nose clips: alcohol swab<br>Remove batteries when storing.
	Emphasize not to run water through turbine.
	Who to contact for troubleshooting or future questions: **Pulmonary Lab**
	Discuss protocol for patient use (5 days initially, sick visits, and always bring to clinic)<br>Start day one tomorrow (measure height).
	Instruct how to use measuring tape to obtain height. (Show patient's last height.) ________<br>• Hard floor area to sticker it to the wall? Stand in middle, feet together, heels back against wall, relaxed hands to sides.<br>• 12-in ruler. Stabilize against wall. Top of ear opening and across occipital bone.<br>• Don't move. Lay ruler flat across top of head and line up to the measuring point.
Other?<br>Questions?	Graph:

Meeting Participants: ____
